# The importance of cancer patients' functional recollections to explore the acceptability of an isometric‐resistance exercise intervention: A qualitative study

**DOI:** 10.1002/hsr2.186

**Published:** 2020-09-08

**Authors:** Ferhana Hashem, Kevin Corbett, David Stephensen, Ian Swaine, Haythem Ali, Irena Hutchins

**Affiliations:** ^1^ Centre for Health Services Studies University of Kent Canterbury UK; ^2^ Centre for Critical Research in Nursing and Midwifery Middlesex University London London UK; ^3^ Kent Haemophilia & Thrombosis Centre East Kent Hospitals NHS Foundation Trust Canterbury UK; ^4^ School of Human Science, Faculty of Education, Health and Human Sciences University of Greenwich London UK; ^5^ General and Upper GI surgery & Musculoskeletal Outpatient Physiotherapist Therapy Services Maidstone and Tunbridge Wells NHS Trust Tunbridge Wells UK

**Keywords:** abdominal cancer surgery, isometric‐resistance exercise, patient identity, patients' functional recollections, physical activity, recovery experiences

## Abstract

**Background and Aims:**

Although it has been widely recognized the potential of physical activity to help cancer patients' preparation for and recovery from surgery, there is little consideration of patient reflections and recovery experiences to help shape adherence to exercise programs. The aim was to explore the acceptability of our newly proposed isometric exercise program in a large general hospital trust in England providing specialist cancer care by using patient recollections of illness and therapy prior to undertaking a randomized controlled trial.

**Methods:**

Four Focus groups (FGs) were conducted with cancer survivors with an explicit focus on patient identity, functional capacity, physical strength, exercise advice, types of activities as well as the timing of our exercise program and its suitability. Thematic framework analysis was used with NVivo 11.

**Results:**

FG data was collected in January 2016. A total of 13 patients were participated, 10 were male and 3 were female with participants' ages ranging from 39 to 77. Data saturation was achieved when no new information had been generated reaching “information redundancy.” Participants reflected upon their post‐surgery recovery experiences on the appropriateness and suitability of the proposed intervention, what they thought about its delivery and format, and with hindsight what the psychological enablers and barriers would be to participation.

**Conclusion:**

Based upon the subjective recollections and recovery experiences of cancer survivors, isometric‐resistance exercise interventions tailored to individuals with abdominal cancer has the potential to be acceptable for perioperative patients to help increase their physical activity and can also help with emotional and psychological recovery.

## 1 INTRODUCTION

Annually, nearly 50 000 United Kingdom patients undergo abdominal cancer surgery with the commonest types of cancer including colorectal, liver, pancreatic, kidney, renal, stomach, ovarian, and cervical cancer.^1^ This group of patients often experience complications that necessitate readmission to hospital requiring high dependency or intensive care, suffer post‐operatively, and longer‐term, many patients experience weight loss and muscle atrophy.^2^ In the United Kingdom, there is no consistent information or advice provided to such patients prior to and following abdominal surgery, in order to mitigate a period of decreased health, combined with fatigue, functional problems, raised anxiety, and limitations in social life, culminating in an overall reduction in quality of life over a prolonged period of time.^3^, ^4^ Currently interventions drawing on patient reflections and recovery experiences from abdominal cancer surgery remain limited indicating a need to consult cancer survivors when introducing new exercise interventions drawing from their subjective recollections as patients to help shape adherence of future health care interventions. The aim of the study was to explore the acceptability of our newly proposed isometric exercise program by using patient recollections of illness and therapy prior to undertaking a randomized controlled trial.

There is a voluminous body of literature on the potential of exercise to help cancer surgery patients' preparation for and recovery from surgery, by minimizing the effects of muscle loss through exercise training.^5^, ^6^, ^7^, ^8^ The effect of strength training, as highlighted by Bergenthal et al, alongside physical activity has the potential to increase mobility and function to aid cancer recovery.^9^ Yet, this literature has been limited in terms of scope and focus with findings on rehabilitation programs being reported on a range cancer types, rather than associated specifically to abdominal cancer.

In recent years, the findings on cancer treatment and exercise have been more nuanced with consideration on cancer type. Hijazi et al,^10^ systematic review on prehabilitation for patients undergoing major abdominal cancer surgery, found that it was unclear what the optimal composition of what programs should consist of, how they should be delivered and what outcome measures should be used to evaluate such programs. Beck et al^11^ examined patients' ability to prepare themselves for major abdominal surgery through a prehabilitation program and found that in order to understand patient compliance, prehabilitation regimes needed to take into consideration patient perspectives to enhance patient‐centredness and adherence. De Almeida et al^12^ found in an early mobilization program following abdominal surgery that performance of exercise activity amongst patients was rather heterogeneous with many partially completing the exercises in the first postoperative days. Colorectal cancer surgery and recovery programs have also been reported in the literature providing a further adjunct to studies on abdominal cancer surgery and physical activity.^13^, ^14^ What remains unanswered are the factors that go beyond physical performance, therefore consideration should be given to tailoring exercise interventions that take into account individual physical activity levels, attitudes toward exercise willingness and preferences through a deeper understanding in relation to adherence.^15^

Our participants underwent focus group (FG) exploration of their perioperative recollections of self‐efficacy to undertake an isometric‐resistance program in order to prospectively inform our RCT evaluating physical function improvement after cancer surgery (forthcoming). Recent emphases on patient recollections show great utility for planning and undertaking clinical trials.^16^, ^17^ The work by Lindberg et al^16^, ^17^ on breast cancer survivors' recollections of their illness and therapy indicate how understanding subjective experiences and recollections need to be considered in patient care, as former patients shape communication about an illness and about the acceptance of health care interventions. We have used a similar approach drawing on the recollections and memories of abdominal cancer survivors to remember their past treatment experiences to explore the acceptability of an isometric‐resistance intervention.^16^, ^17^

## 2 METHODS

### 2.1 Setting and participants

In December 2015, we purposively recruited patients from a large general hospital trust in England providing specialist cancer care for focus group (FG) participation in January 2016, on the basis of experiencing open/laparoscopic abdominal surgery for cancer. The inclusion criteria included all patients who had undergone open or laparoscopic abdominal surgery for cancer in the last 24 months. The exclusion criteria included: patients who were unable to give informed consent or did not have the mental capacity to consent; patients who were undergoing further emergency procedures; and lastly, patients who were undergoing operations which were scheduled in less than 2 weeks' time and therefore receiving urgent care.

### 2.2 Sampling

Our FG method anticipated variation in the number of purposively sampled participants^18^ who were selected as they possessed information rich knowledge of the requisite cancer operation experience.^19^ In addition to knowledge and experience, they were available and willing to take part, as well as able to communicate their experience and opinions in an expressive and reflective manner.^20^

### 2.3 Procedures

A research nurse (MG) approached 25 potential participants face‐to‐face and over the telephone selected via a hospital registry in December 2015. Patient anonymity was maintained as only the research nurse had access to patient contact details available on the registry. An invitation letter and an information sheet were sent by a research nurse and received by former patients before commencement of the FGs outlining the study purposes and aims. Five people approached were unwilling to take part, and five people indicated that they were unavailable. Two further participants who agreed initially did not attend a FG session indicating last minute changes to plans. Due to patient recruitment taking place in the weeks leading up to winter closures few volunteers came forward to take part, so the recruitment criteria for the FGs was widened to include esophageal patients. Written informed consent was obtained from all participants. Ethical approval was granted by the UK's Health Research Authority's National Research Ethics Service.

### 2.4 Conduct of focus groups and data collection

FGs were led by a male researcher (KC) and female research physiotherapist (IH) with two to four participants in each group, located in the hospital treatment centre. KC is an experienced qualitative researcher and Senior Lecturer in Adult Nursing with a 35 year research portfolio working on patient experiences in health and social care. The topic guide was developed by the research team based upon two completed systematic literature reviews,^21^ which identified the functional challenges experienced by patients undergoing elective abdominal cancer surgery so as to inform a deep discussion of our proposed intervention. The FG topic guide included the following: (a) welcome and introductions; (b) review of the aims of the focus group; (c) agree/amend the set of ground rules; (d) qualitatively explore the suitability of the exercises according to the patients' pre and post‐operative experiences, and discuss potential of continuing in the long‐term; (e) finish with other discussion points^2^, ^22^, ^23^, ^24^, ^25^ (Data S1). A FG topic guide was piloted on the project's Patient and Public Involvement members (n = 4) who had undergone the requisite operative experience. Feedback on relevance, comprehension, clarity, and consistency were incorporated in order to refine the topic guide.^19^, ^26^ At the beginning of each FG, the researcher and research physiotherapist introduced themselves and explained their reasons for their involvement in the study. Whilst the researcher had no prior relationship with the participants, the research physiotherapist had met them on previous occasions as was involved in physiotherapy treatment in their post‐operative recovery. Only the researcher, research physiotherapist and participants were present at the FGs. Handouts of the proposed exercise program were circulated within each FG illustrating each different exercise per stage with narrative descriptions each <5 sentences long; see Table 1. In total, the discussion topics took up 80% of FG time. FGs were between 90 and120 minutes long, audio recorded and transcribed at a later date. No other forms of data in the form of video recordings or field notes were undertaken during or after the FGs. Each FG was guided by the use of a semi‐structured topic guide to ensure open‐ended, flexible and spontaneous and in‐depth responses to participants' issues and full thematic exploration mutually between participants and researchers. Repeated FGs were not conducted to reduce participant burden and avoid participant fatigue. Data saturation was achieved when no new information had been generated from the FGs, as individual participants did not express any novel ideas or points thereby reaching “information redundancy.”^27^

**Table 1:** Summary outline of the isometric muscle‐strengthening exercise program

Four stages	Stage 1–4: body areas exercised	Each stage
Stage 1—Before the operation	Abdominal muscles Arms and shoulders Trunk legs Hand and arm Foot and lower leg Abdominal muscles Arms and shoulders Trunk and legs Hand and arm Foot and lower leg	Ten variable muscle‐strengthening exercises which vary per stage (eg, *for “trunk and legs”: actual muscle differ for each stage 1,2,3, and 4*)
Stage 2—The first 2 weeks after discharge home		
Stage 3—From 2 to 6 weeks after discharge home		
Stage 4—From 6 to 12 weeks after discharge home		

### 2.5 Data analysis

FG transcripts were analyzed by two researchers (KC, FH) using a thematic analysis framework^28^, ^29^ and coded electronically using NVIVO 11 (Qualitative Software and Research Pty Ltd.).^30^ Transcripts were not returned to the participants for comment, correction, or feedback due to the difficulty in separating individual responses from collective focus group data. Analysis was derived from the data, and involved familiarization with the transcripts, identification of key themes, indexing data (highlighting quotes/comparing to participants), charting/mapping quotes according to the identified themes and interpretation with reference to the context, with both researchers mutually checking indexing for internal consistency, frequency, and extensiveness of statements/specificity of comments.^29^, ^31^ In order to ensure reliability and validity, we used the strategies developed by Guba and Lincoln associated with credibility in qualitative research to enhance: truth value (through peer debriefing to uncover bias and audio‐recordings of FGs to cross‐check emerging themes); consistency and neutrality (documenting the research process using transparent and clear descriptions, and discussing emergent themes in the team); and applicability (providing rich descriptions of context to evaluate transferability to other settings through guest contributors at research team meetings).^32^, ^33^ Our analysis enabled an exploration of the respondents' discursive recollections of their capacities for perioperative exercise, their foci on the mind and/or the body and the discursive emergence of perioperative operative identities.

### 2.6 Ethical considerations

Ethical approval was obtained from the UK's Health Research Authority's National Research Ethics Service Committee (NRES Committee London— City and East; REC reference 15/LO/0890 granted July 28, 2015). All participants who took part provided informed consent for their participation in the study.

## 3 RESULTS

### 3.1 Participant characteristics

Thirteen different patients participated in four separate FGs: FG1 (n = 3); FG2 (n = 4); FG3 (n = 4); and FG4 (n = 2), respectively. Table 2 shows the characteristics and cancer diagnoses of those 13 participants who were finally included, many of whom were of retirement age, married and/or partnered with a history of elective cancer surgery involving oesophagostomy, laparoscopic prostatectomy, open gastric resection, small bowel resection, abdominal hysterectomy and ovaries. Ten participants were male; three were female with participants' ages ranging from 39 to 77.

**Table 2:** Participants' diagnostic and demographic characteristics

Participant	Sex (M/F)	Operation	Year of Birth
1	M	Oesophagostomy	1960
2	M	Oesophagostomy	1950
3	M	Laparoscopic prostatectomy	1947
4	M	Oesophagostomy	1938
5	M	Right hemicolectomy	1955
6	F	Open gastric resection	1940
7	M	Laparotomy appendicular and bowel resection	1946
8	F	Abdominal hysterectomy and bilateral ovary removal	1976
9	M	Small bowel resection	1948
10	M	Thoraco‐abdominal oesophagostomy	1940
11	F	Laparoscopic anterior resection	1958
12	M	Abdominal approach oesophagostomy	1954
13	M	Ivor Lewis oesophagostomy	1951

### 3.2 Reflections on appropriateness and suitability of proposed intervention post‐surgery

The respondents reflected on the impact of surgery/chemotherapy and the suitability of undertaking an exercise intervention noting diminished perioperative mobility due to surgical drains, catheters, weakness, and an inability to comfortably lie supine. Views converged on how surgery and operative‐type (laparoscopic/open) affected mobility and prospectively influenced individual decisions about undertaking perioperative exercise. The first respondent talked of their body as something autonomous and distinct within the self, whilst the second respondent reported being more severely impeded following surgery:

It wasn't so much I made myself, I felt that my body wanted to get up and do it. I thought I've just got to get up, I've got to walk and I felt comfortable with it. I was very lucky because mine was only laparoscopic which is a big, big difference. I felt amazing, I just felt I feel so well I want to go home now. (Focus Group Four)

Taking into account also because of the complications, I was sent home with an open wound. I had to heal inside out, which didn't help…and I've still got two open wounds from the operation, although I had the operation in October, the wounds haven't healed up. So I have to go to the doctor's surgery every day… (Focus Group Three)

Our participants all acknowledged how exercise ability was functionally dependent on the operation‐type whilst recognizing how structured physical activity had potential benefit. Participants' spoke of a need for an exercise program to assist recovery, reporting how current practice varied, as some received no advice whilst others did:

I was just thinking, you're showing us all these exercises, but we were never asked to do any in hospital…But it seems funny that we weren't given, well, I wasn't personally given this sheet to tell you it would be a good idea to do them when you get home.

I think I might have had a little brochure in my pack. I was given a pack, but because you read it before [surgery]…I didn't think about looking at it after [surgery]. So had I'd been given it like when you get given your [socks] and everything else, or when you're going, then you might take more notice of [it]…Yeah. Or how important it is to recover. (Focus Group Three)

Participants all felt that perioperative information on physical activity was inconsistent. No structured advice was given to aid a return to quotidian “normality,” which was postoperatively severely/suddenly impaired via a rapid transition to reduced mobility. Difficulties were also recollected in accepting functional loss whilst undertaking domestic tasks, bedtime routines and using ramps and stairs, recollecting how such effort preoperatively was once automatic yet postoperatively now required conscious bodily effort:

I just found getting into bed and getting out of bed was just a total nightmare. I actually did an exercise, when I think of it, because I learned a process of getting into bed. (Focus Group Three)

Walking up ramps, if you've ever been to Waterloo Station, you've got that ramp at the top…it took me four attempts to get up there… Once you get to the top of you go out across the bridge…and then down all those steps…So it's ramps, it's stairs, it's stamina, those sorts of things you've got to think about. (Focus Group Two)

Participants agreed that an exercise intervention were needed to help with quotidian functional recovery being surprised that none existed. Of explicit importance was that any newly adopted program must take into account different operation‐types, subsequent recovery times and baseline levels of physical activity (see final theme).

### 3.3 Responses to the delivery and format of the intervention

Participants recollected their pre‐operative experience and the exercises that professionals advised to do. They reflected on how they acted on this advice in order to prepare themselves before surgery to enhance their recovery. It is noticeable how some participants recollected consciously guiding (easing) bodily behavior. Others spoke of the body as a distinct entity in relation to weakening due to chemotherapy yet also in terms of perseverance:

Well, no, to be truthful, I didn't start it until XXXXX said about getting fit for this hernia operation. He said he wanted me to be as fit as I could, so walking was a good thing, so I thought, right, that's what I'd try and do every day… (Focus Group Three)

It left me very weak and very exhausted but the body recovers quickly. So the exercise regime that I was given was very much based around the exercise bike. I did a lot on the exercise bike at home to build up the stamina, from a couple of minutes to three minutes to four. So by the end of three weeks of feeling strong enough to get on it, being told what to do and then surgery, I could go from two minutes on the exercise bike to twenty… (Focus Group Two)

Participants all felt they would be able to commence a structured program after surgery. One participant recollected how his mental initiation of physical exercise post‐operatively was consciously spurred on by his history of sports‐training: The first participant felt he could have considered exercising 2 to 3 weeks post‐operatively, whilst others required a longer recovery period without contemplating exercise. The second participant recounted a cumulative approach to post‐operatively achieving what they recollected was their pre‐operative level of lateral functionality:

…I found any exercise, apart from the first fortnight, three weeks say, but once I started doing something, I found it was fine…Oh yeah. With my background in sport, I thought I've got to get moving and I was doing sit ups after a fortnight, did a few press‐ups.

(Focus Group Four)

So really for the first six weeks, I could only use my left [side], and my wife is heavily into yoga, and she gave me all sorts of different exercises to use and I gradually built‐up and built‐up and over the course of a year, I got the use of that back again.

(Focus Group One)

Some participants were also able to set/reset their own individuals exercise goals depending on performance self‐appraisal using a cumulative approach similar to the above:

…I too did a lot of walking and I think that was the main form of exercise that I had. Anytime I felt that I achieved my goal and I'd walked 50 yards, then I extended it to another 50 and so on and so forth until I was walking up to 3 miles without any problem. That was significantly after 2 weeks. (Focus Group Two)

In terms of a suitable format, participants felt that an induction followed by a structured portable home‐based program and weekly contact with a physiotherapist for adherence would be beneficial:

*…*the physio…he just came in and set the same exercise for everybody and it was just three movements and he said, “Well you can already do them so that's fine,” but yes, …but if you got a set formula and you come home with something… (Focus Group One)

Maybe if you had one or two sessions while you were in hospital, at least you would know then what you could do. (Focus Group Three)

### 3.4 Psychological enablers/barriers to participation

#### 3.4.1 Enablers

Participants spoke of inducements to encourage participation, especially noting their desire to return to feeling “normal.” The statement below shows how exercise was associated with walking which made the respondent feel “normal” again, thus the expectation of any program was aiding a return to the “normal self.”

I was very conscious of getting back to normality. With me, because I'm not a particularly athletic person in any shape or form, the only thing that I do is walk, I love being out in the open elements and I love to walk. (Focus Group Four)

Others' sense of feeling “normal” entailed returning to their relatively higher levels of physical activity. This emerged in discussions as an athletically trained patient who underwent surgery had the goal of returning to his sense of feeling *like* he *had felt prior to surgery* in terms of pre‐surgical activity baseline, or identity (*“ingrained in your psyche”*) and in relation to the aging processes. It resembles an ingrained tactile sensation which is recollected from the pre‐operative memories of physical functionality, a recollection of one's pre‐operative identity. He recollected that he:

XXXXX was actually pretty programmed really because that's been his way of life, however long it was, 20 years so you don't go through that sort of practice without it being ingrained in your psyche. (Focus Group Four)

The participants talked of overcoming the psychological trauma of surgical recovery and keeping a positive frame of mind but in terms of the importance (“*90 %”*) of exerting cognitive bodily control for prospective recovery *(“get on with it”*); a lay phrase redolent of the maxim *mind over matter*. This was also associated with goal setting/achievement as a personal method/yardstick for measuring or regulating one's own degree of improvement:

Ninety percent of recovery is in here [points to head] and at the end of the day you want to get on with it you know… You got to fight on and go for it… I didn't fall into a heap on the floor and cry… (Focus Group One)

You do find it harder, but if you've got something to measure your progress with yourself that's an everyday thing then, again, mentally you're finding you're doing something… I can measure it by how much water I can put in the kettle, or water in the pan on the cooker. (Focus Group Three)

Setting and building upon realistic goals was an important consideration for sustaining motivation levels. This again points to the mind (*“mental thing”*) consciously aiding sensate improvement (*“feel better in yourself”*).

That's right, and it's to build on that. And it goes back to it's a mental thing as well. You've got to set yourself some goals and targets. And they mustn't be stupid. They've got to be sensible. And that's what you strive to achieve. And every time you do it, it's a success, and you feel better in yourself. (Focus Group Three)

Others felt that designing any exercise intervention must take account of varying age/ability to ensure patient motivation. Thus, a more “performative” type of design may more likely “fit” different bodies rather like a shoe is designed to fit different types of feet and be “tried on” beforehand in order to judge/help gauge the “fit”:

It'd be good if you could actually have maybe three different sets of different types of exercises aimed at different age groups and see whoever fits into them. (Focus Group Four)

#### 3.4.2 Barriers

Participants discussed how post‐operative movement was limited, impacting mobility, and subsequent ability to reflexively engage the body in undertaking physical activity. Others spoke of feeling sick and exercise being the furthest from their mind:

Well, no it's again it's a slight annoyance but I mean I was agitated, it's almost if you get up and go as much as I could, the drains… I had the more I started to getting out of bed and moved around so… I was thinking, “Get up, you can get up and walk, do something.” (Focus Group One)

Yes, I can just remember being tired but I can't remember ever thinking about anything that I did in the way of exercise. I don't know. Because I just felt sick all the time, I just felt sorry for myself. (Focus Group Three)

One participant reflexively spoke of frustration/spousal dependency as spurs to bodily action. There was a consensus that some patients were willing dependency and not proactive with physical recovery, a tacit reality which was “called out” as above by one patient to the other as “peers.” One respondent spoke of lack self‐motivation as a barrier to exercise:

…a chap I know in XXXXXXXX, he had a hip operation and he came out of hospital and a physio came round. I said to him what did the physio say, did he give you any exercises and he said yeah. He pushed his toes out straight, brought them back up again and then put his leg down. I said yeah, didn't he ask you to move your hip and he said no. I said how many times have you got to do that, he said twice a day. I said what, just once? He said yeah. I said I'm sorry, I don't believe you. I said if you don't get up and start walking with your crutches and everything, you're going to be stuck in that chair. (Focus Group Four)

Participants spoke of their performance of a particular physical movement as being a reminder of their current restricted levels, which had not yet returned to their pre‐operative mobility. Not being able to perform simple everyday tasks was also reportedly disempowering:

And getting in and out of the car was difficult. That was difficult. I suppose it depends on what type of car you've got, but yeah, I think it depends on what type of operation you've had. It's a simple thing but it was actually quite disappointing. (Focus Group Three)

The effects of surgery/chemotherapy had an immense emotional impact on participants' post‐operative identity referred to as “*becoming back to who you were*,” an explicit recollection of an influential pre‐operative identity. It suggests how feeling able to overcome emotional barriers is a key to patients considering performing a new physical task in an exercise program.

## 4 DISCUSSION

The key messages from the results indicate that operation‐type, post‐surgery recovery experiences, and the impact on mobility all influence acceptability of an isometric‐resistance exercise intervention in abdominal cancer surgery patients. How participants recollected their perioperative selves was notable in terms of the different roles and emphases discursively ascribed by participants reminiscent of a Cartesian‐like duality of the “mind” and the “body.” There were some data suggesting that ingrained tactile sensations are recollected from these pre‐operative memories of physical functionality, a form of bodily‐know‐how, or stored habituated behavior, which some schools of educational theory suggest may be helpful for optimum task performance.^34^

Variable and patchy exercise advice to enhance patient recovery was also a notable finding. The participants were dismayed that exercise advice was inconsistent perhaps reflecting an existing lack of robust evidence on the effects of physical activity on post‐operative cancer recovery. Having access to recommended advice and information on post‐surgery exercises has been noted by Gupta et al^35^ as an important consideration for patients' recovery. He found that patients in his study were receptive to being given age‐specific brochures, relevant references to web pages, and information on local exercise programs and walking activities that contributed to their sense of empowerment and helped to reenter normal like. Our participants clearly wanted a reliable regimen based on recommendation to encourage in their functional recovery.^35^

We also found that participants reflected on the efficacy of undertaking perioperative prehabilitation exercise at home. We found that participants who had been advised to undertake home‐based exercises reported being able to perform them following surgery as instructed even though when fatigued. This mirrors Chen et al's^36^ findings who report upon a user‐friendly home‐based prehabilitation program who found higher levels of adherence and longer functional maintenance rates with home‐based programs. A home‐based setting was commented by our participants as beneficial for facilitating the ease of exercising once back in a familiar stable environment.

Despite the availability of online formats, the participants stated a preference for a face‐to‐face intervention with weekly practitioner contact. Rabin et al^37^ reported that cancer survivors preferred in‐person interventions especially those which required behavior change such as exercise/walking or yoga classes, as they offered an opportunity for developing better social connections with trainers. Professional oversight is reportedly imperative to ensure sufficient progression through any training process,^38^ rather than traditional interventions for cancer survivors that have had a “one‐size‐fits‐all” design.^39^ Our participants felt that professional oversight was key for adherence and that a “one‐size‐fits‐all” program was inappropriate, preferring an intervention tailored to different levels, abilities and ages, with progress measured in relation to individual goals/baselines in a self‐selected manner.

The enablers/barriers identified in the findings focused on psychological considerations that either helped or hindered exercise participation. Participants reported wanting to return to feeling “normal,” recollecting their pre‐operative selves/identities, and if the prospect of taking part in an exercise regime would enable them to return to their pre‐operative sense of “normality,” then they felt it would be worth participating. These were reflecting feelings of weakness/vulnerability in the context of believing they had grown post‐operatively to be somehow different from their earlier “selves.” Cancer‐diagnosed athletes engaging in an exercise intervention are known to start feeling “normal” even after cancer therapy, as the exercise environment fosters both the self‐realization of a functioning body with reserve resources and a positive self‐identity.^40^ For the athletes in Adamsen et al's study, exercise provided a platform for participants to reclaim a sense of self‐identity and bodily control through exercise participation. Similarly, our participants reported expectations that an exercise program would help to restore their sense of personal and physical identity perhaps akin to their pre‐operative selves.

The barriers to exercise participation related to limited mobility and a concomitant impact on post‐operative/post‐chemotherapy motivation, all of which reportedly caused low morale/frustration not uncommon in perioperative cancer patients.^38^ Given the known rate of anxiety/depression within our demographic, supporting interventions focusing on physique/mental well‐being to help negate this morbidity is advised.^38^ It has also been noted in other studies by Sjösten and Kivelä^41^ and Mammen et al^42^ that cancer survivors welcomed the prospect of taking part in an exercise intervention to help improve their physical, emotional, and psychological recovery.

### 4.1 Limitations

The patients invited to take part in the FGs varied in terms of age, sex, and self‐reported physical fitness, with some in their late seventies and others in their forties with variable experiences of exercise. This may limit the transferability of the findings to all patients. From the FGs, participants had differing views on what they conceived as physical exercise, with some indicating walking, while others perceived cardio‐vascular exercises as the main forms of activity. The FG discussions encouraged participants to recall their functional mobility post‐surgery and to reflect upon whether they would be able to perform regular exercises, yet any negative experiences may have created a recall bias in gauging whether a program would be acceptable.

### 4.2 Clinical implications

The subjective experiences of cancer survivors may help with healthcare professionals' understanding of the nonclinical side effects of cancer treatment relating to post‐operative recovery, physical mobility, as well the psychological distresses remembered by patients. Professional oversight of exercise programs by trainers, as suggested could encourage adherence, with bespoke tailored interventions taking into consideration different levels, abilities and ages. These factors could be key to encouraging acceptability of an exercise program.

## 5 CONCLUSION

Our study showed that based upon the subjective recollections and recovery experiences of cancer survivors, an isometric exercise intervention tailored to individuals with abdominal cancer has the potential to be acceptable for perioperative patients to help increase their physical activity, as well as helping with emotional and psychological recovery. A structured isometric‐resistance exercise intervention was welcomed, one which was professionally guided/tailored in hospital to individual functional capacity to help improve safe quotidian home recovery. The enablers/barriers to program engagement included psychological factors influencing exercise adherence and self‐efficacy to safely perform exercises given the psychological distress associated with surgical cancer treatment.

## FUNDING

This paper presents independent research funded by the National Institute for Health Research (NIHR) under its Research for Patient Benefit (RfPB) Programme (Grant Reference Number PB‐PG‐0613‐31107). The views expressed are those of the author(s) and not necessarily those of the NIHR or the United Kingdom's Department of Health and Social Care. The NIHR RfPB programme did not have any involvement in the study design, data collection, analysis, interpretation of the data, writing of the report, or the decision to submit the report for publication.

## CONFLICT OF INTEREST

The authors declare no conflicts of interest.

## AUTHOR CONTRIBUTIONS

Conceptualization: Ferhana Hashem, Kevin Corbett, David Stephensen, Ian Swaine, Haythem Ali, Irena Hutchins

Data curation: Ferhana Hashem, Kevin Corbett

Formal analysis: Ferhana Hashem, Kevin Corbett

Funding acquisition: Ian Swaine, Haythem Ali, Ferhana Hashem, David Stephensen

Investigation: Kevin Corbett, Irena Hutchins

Methodology: Ferhana Hashem, Kevin Corbett

Project administration: Ian Swaine, Haythem Ali, Ferhana Hashem, Kevin Corbett, David Stephensen

Resources: Kevin Corbett, Ferhana Hashem

Software: Ferhana Hashem, Kevin Corbett

Supervision: Kevin Corbett, Ferhana Hashem

Validation: Ferhana Hashem, Kevin Corbett

Visualization: Kevin Corbett, Ferhana Hashem, Ian Swaine, Haythem Ali, David Stephensen

Writing – original draft preparation: Ferhana Hashem, Kevin Corbett

Writing – review and editing: Ferhana Hashem, Kevin Corbett

All authors have read and approved the final version of the manuscript.

Ferhana Hashem had full access to all of the data in this study and takes complete responsibility for the integrity of the data and the accuracy of the data analysis.

## TRANSPARENCY STATEMENT

Ferhana Hashem affirms that this manuscript is an honest, accurate, and transparent account of the study being reported; that no important aspects of the study have been omitted; and that any discrepancies from the study as planned (and, if relevant, registered) have been explained.

## Supporting information


**Data S1**. Supporting Information.

## Data Availability

The data that supports the findings of the study are available on request from the corresponding author. The data are not publicly available due to privacy or ethical considerations.
