# Supplementary material for: The importance of cancer patients' functional recollections to explore the acceptability of an isometric‐resistance exercise intervention: A qualitative study
Source: Health Sci Rep. 2020 Sep 8;3(3):e186. doi: 10.1002/hsr2.186 (PMC7507341; doi:10.1002/hsr2.186)
Supplement: Supplementary file 1 — Data S1. Supporting Information. [file HSR2-3-e186-s001.doc]

**A study to develop an isometric resistance-exercise programme (EPOP1) for cancer patients to improve recovery after cancer surgery:**

**Focus group: ground rules for participants**

Aim of the focus group:

To gain a deeper understanding of what the challenges are to physical functioning experienced by patients following cancer surgery (including abdominal surgery), which might be addressed by an exercise programme.

1. Listen actively and respect others when they are talking.
2. Speak from your own experience ("I" instead of "they," "we," and "you").
3. Do not be afraid to respectfully challenge one another by asking questions, but refrain from personal comments, and concentrate on ideas.
4. Participate to the fullest of your ability. Optimum participation depends on the inclusion of everyone.
5. Share your own story and experiences about physical abilities and functioning.
6. During the focus group you are free to disclose personal information but do not repeat outside of the focus group any personal information that may have been disclosed by others.

**Focus Group Topic Guide**

| **Agenda item** | **Time [approx.] [max. 150 minutes]** | **Facilitator behaviour** |
| --- | --- | --- |
| 1. Welcome and introductions. | [15 minutes max.]  5 mins  10 minutes  10 minutes | *Icebreaker*:  Each facilitator briefly introduces themselves  *If equal number of participants*: Ask participants to pair up & briefly introduce themselves to their partner; then each participant within each introduces the other partner  *If unequal number of participants*: ask each In turn to introduce themselves |
| 1. Review the aims of the focus group | 5 minutes | Review the FG aims & ground rules  Establish trust and demonstrate respect through appropriate verbal and non-verbal behaviour  Answer any questions |
| 1. Agree/amend the ground rules. | 5 minutes | Elicit feedback  Amend/agree ground rules |
| 1. Review the feasibility of the assembled exercises according to their applicability to the following phases of the patient experience :  - *Preoperative*- Stage 2 & 3 Exercise Programme - *Postoperative*- Stage 1 Exercise Programme - *Postoperative and The Longer Term*- Stage 2 & 3 Exercises Programme | 35 minutes    35 minutes  35 minutes | Check and/or explore understanding  Identify and/or explore novel approaches and/or understandings  Aim to ensure equity of participation |
| 1. Any other discussion points | 10 minutes | Elicit responses checking and/or exploring understanding  Identify and/or explore novel approaches and/or understandings  Aim to ensure equity of participation |
| 1. Close | 5 minutes | Thank participants for their input |
